# A rare case presentation: pregnancy and gastric carcinoma

**DOI:** 10.1186/s12876-020-1184-9

**Published:** 2020-02-12

**Authors:** Mustafa Yildiz, Yesim Akgun, Hale Ozer, Veli Mihmanli

**Affiliations:** Health Sciences University Okmeydani Training and Research Hospital, Obstetrics and Gynecology Clinic, Istanbul, Turkey

**Keywords:** Gastric carcinoma, Pregnancy, Endoscopy

## Abstract

**Background:**

Gastrointestinal system (GIS) malignancy with pregnancy is a very rare condition and is not common outside Japan. The incidence is between 0.025–0.1% for each pregnancy. GIS malignancies are diagnosed late in pregnancy and detected at an advanced stage. The most common cause of this condition is that the symptoms such as vomiting, nausea, loss of appetite and abdominal growth are mistaken with pregnancy and malignancy is overlooked. Especially in the second trimester, symptoms such as nausea and vomiting, weight loss, melena, hematemesis and deep anemia should suggest malignancy. Upper GIS endoscopy and colonoscopy are the recommended screening methods in these patients, especially in the third trimester.

**Case presentation:**

We present a rare case presenting to our emergency room with the complaint of bloody vomiting, at the 36th week of gestation with a live singleton pregnancy, and receiving the diagnosis of undifferentiated gastric carcinoma from the biopsy taken from the ulcerated lesion on the stomach cardia, with upper GIS endoscopy performed due to deep anemia, who underwent simultaneous cesarean section and subtotal gastrectomy.

**Conclusion:**

Gastrointestinal system (GIS) malignancy with pregnancy is a very rare condition, but it should be considered when symptoms such as nausea and vomiting, weight loss, melena, hematemesis and deep anemia occur, especially in the second trimester, and endoscopic screening should be recommended. Because of the delay in diagnosis of malignancy and the detection in advanced stages, patients should be referred for treatment without delay.

## Background

Gastric cancer detected during pregnancy is a very rare condition and is usually diagnosed in advanced stages with a poor maternal and fetal prognosis [1]. Gastric cancer is often seen in older age and is twice as common in men as in women, however it is more common in women in younger patients [2]. Gastric cancer diagnosed during pregnancy or lactation occurs in a rare probability of only 0.025 to 0.1% of all pregnancies and the majority of these cases have been reported in Japan [3]. We report a rare case of undifferentiated gastric carcinoma diagnosed at the 36th week of gestation, who underwent cesarean section and subtotal gastrectomy at the same time.

## Case presentation

**Fig. 1:**
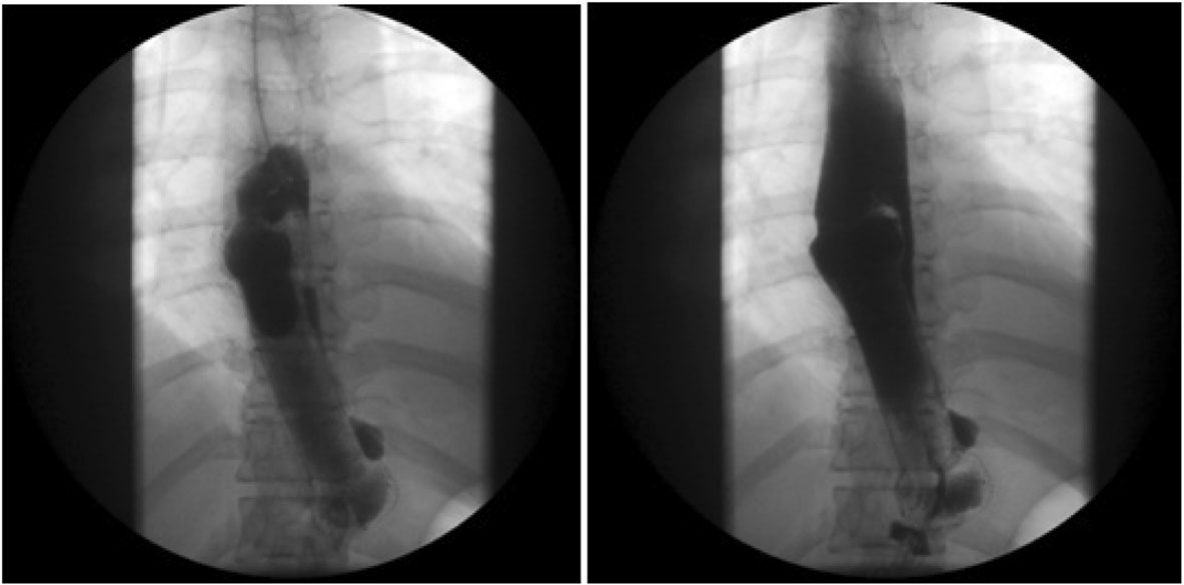
Pictures of endoscopic intervention. İmages obtained during the endoscopy procedure performed during the diagnostic research

A 44-year-old female patient was in the 36th week of her second pregnancy. She was admitted to the Emergency Department of Okmeydani Training and Research Hospital of Health Sciences University with the complaint of bloody vomiting for 2 days. In her deepened anamnesis, it was learned that she had black defecation for 1.5 months and epigastric pain since the beginning of pregnancy. It was also learned that the patient was receiving antihypertensive treatment for hypertension known before pregnancy and was using insulin for gestational diabetes mellitus. The hemoglobin level was found to be 6.4 mg/dL and the patient was admitted to the Internal Medicine Department with gastrointestinal system (GIS) bleeding. Because she was pregnant, she was asked to consult with our Obstetrics and Gynecology clinic. After evaluation with ultrasound (US) and Non-Stress Test (NST), fetal heartbeat (FHB) positive pregnancy compatible with 36 weeks was found and no obstetric pathology was not considered. Endoscopy for GIS bleeding revealed bleeding from Forrest 1A ulcer lesion in the gastric cardia and bleeding was stopped by sclerotherapy (Fig. 1).

A total of 4 units of erythrocyte suspensions were transfused and the patient was discharged after clinical and laboratory follow-up. One week after discharge, the patient was re-admitted to the Emergency Department with the complaint of deterioration in her general condition and bloody vomiting. After the interventions performed, fresh blood came from the nasogastric catheter and the bleeding could not be stopped by endoscopy, the emergency surgery decision was taken by the General Surgery team, we were informed because the patient was pregnant and a joint operation plan was made. The operation was started with a median incision above and below the umbilicus and a 2455 g female baby was delivered, live and coming foot first, with a 1st minute APGAR score of 8, by cesarean section. The patient was then transferred to the general surgery team with the abdomen open. Subtotal Gastrectomy and Liver Wedge Resection were performed. The patient was discharged after having no problems in post-operative follow-up. Her baby was discharged after 10 days of follow-up in the Neonatal Intensive Care Unit. When the pathology report resulted in undifferentiated carcinoma (CerbB2 negative) and liver serosal surface tumor infiltration, the patient was referred to Medical Oncology and chemotherapy and radiotherapy sessions were initiated. During this 6-month period, the treatment is on going.

## Discussion

Symptoms such as nausea, vomiting, loss of appetite may also occur during the natural course of pregnancy and may cause delays in gastric cancer diagnosis. The delay in diagnosis affects the prognosis and stage of gastric cancer. In a study conducted in 2009, 92.5% of diagnosed patients had advanced stage and poor prognosis, and their 1 and 2-year survival were 18 and 15.1%, respectively [3]. In a series of cases in which Sakamoto et al. examined 137 patients, 100 of whom were in Japan, this delay was attributed to four reasons [3]. The emergence of symptoms similar to common symptoms in pregnancy, such as vomiting, dyspepsia and increased abdominal size; the support of neoplastic cells by estrogen in pregnancy; the prevalence of Helicobacter pylori in pregnancy; and the increased blood circulation in pregnancy contribute to the late and advanced stage detection of GIS malignancies in pregnancy [1, 3–5]. Especially in the second trimester, symptoms such as nausea and vomiting, weight loss, melena, hematemesis and deep anemia should suggest malignancy. Because of this, early gastroscopy can be suggested to patients at risk [6]. Without these findings, the indication for performing upper GIS endoscopy is controversial. Upper GIS endoscopy, which is the preferred method for diagnosis in these cases, is considered a low-risk procedure during pregnancy and should not be delayed when indicated, but it is recommended that it be performed in the second trimester of pregnancy if possible [6].

Clinical and experimental studies have shown that the occurrence and development of gastric cancer in women is related to biological and hormonal factors [7]. Estrogen hormone predominance is thought to contribute to the development of neoplastic cells, and 55.8% of gastric tumors have been proven to be positive for estrogen receptor in clinical studies [8]. Estrogen receptors (ER) are found in about 22% of gastric cancer cells, especially the undifferentiated type [1].

Standard interventions in cancer diagnosis, staging and treatment can be harmful to the fetus. Treatment of cancer in pregnancy should not be significantly different from treatment in non-pregnant women [9]. In the literature, there are case reports of pregnancy and gastric cancer cases with complications such as perforation and bleeding requiring urgent surgery. In this case, emergency surgical intervention should be done immediately [10]. In our case, endoscopic intervention was tried first because of GIS bleeding but an operation decision was made because of failure. Pregnancy was terminated during the operation because the gestational week was greater than 34 weeks.

Gastrointestinal system (GIS) malignancy with pregnancy is a very rare condition, but it should be considered when symptoms such as nausea and vomiting, weight loss, melena, hematemesis and deep anemia occur, especially in the second trimester, and endoscopic screening should be recommended. Because of the delay in diagnosis of malignancy and the detection in advanced stages, patients should be referred for treatment without delay.

## Data Availability

Patient data and materials used in this case presentation are available in the electronic data warehouse of our hospital. The following links are available. http://okmeydanieah.saglik.gov.tr/ https://okmeydanieah.istanbulsaglik.gov.tr/hastaportal/#/?p=results

## Abbreviations

APGAR: Activity-Pulse-Grimace-Appearence-Respiration
dl: Deciliter
ER: Estrogen receptors
FHB: Fetal heartbeat
GIS: Gastrointestinal system
gr: Gram
mg: Miligram
NST: Non-Stress Test
US: Ultrasound
